# Adverse Events in a Cohort of HIV Infected Pregnant and Non-Pregnant Women Treated with Nevirapine versus Non-Nevirapine Antiretroviral Medication

**DOI:** 10.1371/journal.pone.0012617

**Published:** 2010-09-07

**Authors:** Erika Aaron, Mirjam-Colette Kempf, Shannon Criniti, Ellen Tedaldi, Ed Gracely, Amy Warriner, Ritu Kumar, Laura H. Bachmann

**Affiliations:** 1 Division of Infectious Diseases and HIV Medicine, Department of Medicine, Drexel University College of Medicine, Philadelphia, Pennsylvania, United States of America; 2 Department of Epidemiology, University of Alabama at Birmingham, Birmingham, Alabama, United States of America; 3 Temple University School of Medicine, Philadelphia, Pennsylvania, United States of America; 4 Department of Family, Community, and Preventive Medicine, Drexel University College of Medicine, Philadelphia, Pennsylvania, United States of America; 5 Department of Medicine, University of Alabama at Birmingham, Birmingham, Alabama, United States of America; 6 Department of Internal Medicine, Mercer University, Macon, Georgia, United States of America; 7 WG Hefner Medical Center, Salisbury, North Carolina, United States of America; 8 Wake Forest University Health Sciences, Winston-Salem, North Carolina, United States of America; University of Cape Town, South Africa

## Abstract

**Background:**

Predictors of adverse events (AE) associated with nevirapine use are needed to better understand reports of severe rash or liver enzyme elevation (LEE) in HIV+ women.

**Methodology:**

AE rates following ART initiation were retrospectively assessed in a multi-site cohort of 612 women. Predictors of onset of rash or LEE were determined using univariate and multivariate analyses.

**Principal Findings:**

Of 612 subjects, 152 (24.8%) initiated NVP-based regimens with 86 (56.6%) pregnant; 460 (75.2%) initiated non-NVP regimens with 67 (14.6%) pregnant.

**LEE:**

No significant difference was found between regimens in the development of new grade ≥2 LEE (p = 0.885). Multivariate logistic regression demonstrated an increased likelihood of LEE with HCV co-infection (OR 2.502, 95% CI: 1.04 to 6, p = 0.040); pregnancy, NVP-based regimen, and baseline CD4 >250 cells/mm^3^ were not associated with this toxicity.

**Rash:**

NVP initiation was associated with rash after controlling for CD4 and pregnancy (OR 2.78; 95%CI: 1.14–6.76), as was baseline CD4 >250 cells/mm^3^ when controlling for pregnancy and type of regimen (OR 2.68; 95% CI: 1.19–6.02 p = 0.017).

**Conclusions:**

CD4 at initiation of therapy was a predictor of rash but not LEE with NVP use in HIV+ women. Pregnancy was not an independent risk factor for the development of AEs assessed. The findings from this study have significant implications for women of child-bearing age initiating NVP-based ART particularly in resource limited settings. This study sheds more confidence on the lack of LEE risk and the need to monitor rash with the use of this medication.

## Introduction

The introduction of antiretroviral therapy (ART) has changed HIV management from that of a uniformly fatal disease to that of a chronic disease. Despite significant advances in HIV therapy, ART-associated toxicities remain an important factor in the treatment of HIV-infected persons. ART options, including for pregnant women, are limited particularly in resource-limited settings (RLS) and many countries rely on the use of nevirapine (NVP). Adverse events characterized by liver and skin toxicities, usually occurring within the first 18 weeks of treatment, have been associated with the use of NVP [1]–[4]. Investigators have reported these toxicities more frequently in women with higher baseline CD4 counts (>250 cells/mm^3^) [5]; in pregnancy [6]; and in the presence of pre-existing liver disease (i.e. hepatitis C) [7]. However, it has not been confirmed that pregnancy increases the risk of liver enzyme elevations (LEE) or rash in women receiving NVP or other ART [8]–[10].

Due to the inability to systematically identify severe but non-fatal toxicities associated with ART in pregnant patients outside of clinical trials, the true incidence of adverse events associated with NVP use in pregnancy is unknown [11]. This knowledge is critical for facilitating the decision-making process of the clinician in selecting optimal ART agents for use in pregnant patients.

Utilizing standardized data collected from three large HIV clinics serving HIV-infected women, we sought to assess the prevalence and the predictors of developing LEE and rash associated with NVP as compared to non-NVP regimen use in pregnant and non-pregnant women.

## Methods

### Study Design

Three university-based clinics with large prenatal patient populations participated in a multi-center retrospective cohort study: Drexel University College of Medicine, Philadelphia, PA; Temple University, Philadelphia, PA; and the University of Alabama at Birmingham, Birmingham, AL. This study was approved by the institutional review board at each institution. A database search identified all female patients on ART between January 1999 and August 2005. Women 18–55 years old who initiated a new ART regimen during that time period and had at least one follow-up medical visit within 12 weeks of initiation, or within 12 weeks of their first prenatal care visit, were eligible for study entry. Eligible subjects could not be on ART at the start of the study period. Women who became pregnant while on an ART regimen were excluded. Thus both ART-naïve and ART-experienced women were eligible, but only those women who were not on ART at the entry into the study were included. Women must have been followed for at least 12 weeks and up to 18 weeks after the initiation of a new ART regimen. Women who initiated ART during the prenatal period and thus had any exposure to ART during pregnancy were included in the pregnancy group. Women who delivered during the 12–18 week period were followed throughout the study time frame into the postpartum period. Only the initial regimen that was started in this time frame was included for analysis. Only the first pregnancy was included in the analysis for women with more than one pregnancy during the study period.

Data were checked for inter-rater reliability and entered into a central database. Only charts that had clear documentation of client visits and assessment of side effect profile during the study period were included.

Demographic and medical information included: age, race/ethnicity, HIV transmission risk, HIV disease stage, ART regimens used during the study period, history of drug/alcohol use, liver function tests (LFT), nadir (lowest CD4 count recorded) and baseline CD4 count (result obtained at therapy initiation) as well as CD4%, HIV RNA measures within 4 weeks prior to the initiation of ART and throughout the study period, hepatitis C virus (HCV) status, presence of AE symptomatology, medication allergy, or intolerance.

### Study Definitions

Baseline lab values were defined as measurements taken up to 28 days before regimen initiation. Abnormal alanine aminotransferase (ALT) and aspartate aminotransferase (AST) values and rash were graded for severity according to the Toxicity Tables of the Division of Acquired Immunodeficiency Syndrome (DAIDS) [12]. Subjects were defined as having LEE if they had 1) at least one episode of new grade ≥2 elevation in ALT/AST after one week (considered baseline) and within 18 weeks of ART initiation, or 2) for patients who entered with baseline grade ≥2 elevation, at least one episode of elevation that was greater than their baseline value after one week and within 18 weeks of ART initiation. Patients who had a grade 4 LEE at baseline or had a rash at the time of regimen initiation were excluded. Due to the paucity of hepatitis C virus (HCV) viral load results available in the medical record, a positive HCV serology was considered synonymous with active hepatitis C infection for the purposes of this study. Patients whose rash was caused by other reasons, such as pruritic urticarial papules and plaques of pregnancy, herpes zoster, allergic dermatitis, etc., were excluded from entry into the study.

### Statistical Analysis

The sample size had ≥80% power to detect a difference in adverse events, of 10% vs. 3%, for NVP vs. non-NVP. Statistical analyses were performed using SPSS Version 15. Chi-square tests for dichotomous outcomes were used for regimen comparisons. Analyses were stratified by baseline CD4 cell count (≤250 cells/mm^3^ vs. >250 cells/mm^3^) and pregnancy status. Unpaired t-tests or Mann Whitney U tests were used for continuous variables. Results were considered statistically significant at p≤0.05. Univariate analyses were performed to test the difference in adverse event rates between NVP and non-NVP regimens. The results are expressed as relative risks. Multivariate logistic regression analysis was used to assess possible confounding factors such as pregnancy, baseline CD4 count (≤250 cells/mm^3^ vs. >250 cells/mm^3^), and presence of HCV. Odds ratios (OR) and 95% confidence intervals (CI) are provided. Because of the small number of events and specific research questions, no correction for multiple comparisons was employed.

## Results

### Study Sample

**Figure 1:**
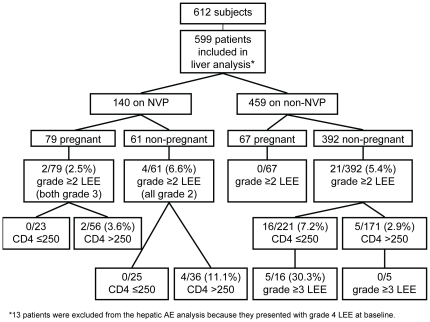
Subjects included in hepatic adverse events analysis.

Of the 612 subjects included in the analyses, 152 (24.8%) initiated NVP-containing ART, 86 (56.6%) of whom were pregnant. Among the remaining 460 (75.2%) patients who initiated non-NVP-containing regimens, 67 (14.6%) patients were pregnant. Thirty-nine women became pregnant after initiating ART and were excluded. Among the pregnant women included 66 started ART at the initiation of the study with baseline CD4 counts below 350 cells/mm^3^ (37 [56.1%] on a NVP-based regimen) and received ART based on their current stage of HIV disease. The remaining 87 women started ART at baseline CD4 counts above 350 cells/mm^3^ (49 [56.3%] on a NVP-based regimen) and initiated ART for the prevention of mother to child transmission (PMTCT). Based on the exclusion criteria [see Study Definitions], 599 women were included in the liver analysis and 526 in the rash analysis. **[** **Figures 1** **and** **2** **]**.

**Figure 2:**
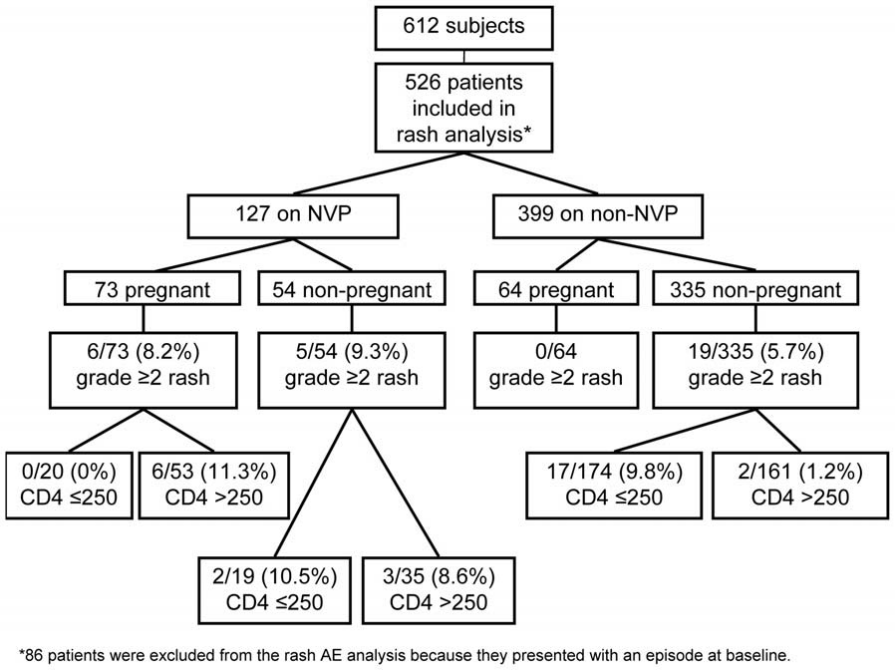
Subjects included in rash analysis.

The baseline demographic and clinical characteristics are shown in Table 1: 72.4% (n = 443) patients were of black race/ethnicity, 18.1% (n = 111) were of white non-Hispanic race/ethnicity, 8.7% (n = 53) were of Hispanic race/ethnicity and 0.3% (n = 2) were multiracial. Three subjects were missing race/ethnic information. The racial/ethnic distribution did not differ between the treatment groups (p = 0.191). Of those with known transmission risk, heterosexual mode of transmission was the primary risk factor in both groups (83.6% for NVP and 85.4% for non-NVP, p = 0.538). NVP recipients had higher baseline CD4 counts, and lower HIV-1 RNA viral loads (both p values <0.001). There was no significant difference in co-infection with HCV (22.0% NVP vs. 17.9% non-NVP, p = 0.328) when stratified by type of regimen use **[** **Table 1** **]**.

**Table 1:** Demographic and Clinical Characteristics at Enrollment for 612 HIV+ Women stratified by Antiretroviral Regimen.

	Subjects on NVP-based regimen (*n* = 152)	Subjects on non-NVP-based regimen (*n* = 460)	*P-value*
**Age (mean; years)**	32.8	37.3	**<0.001**
**Pregnancy**	86 (56.6%)	67 (14.6%)	**<0.001**
**Race**	N = 152	N = 457	0.191
Black	112 (73.7%)	331 (72.4%)	
White, non-Hispanic	31 (20.4%)	80 (17.5%)	
Hispanic	9 (5.9%)	44 (9.6%)	
Multiracial	0	2 (0.4%)	
**Mode of transmission**	N = 140	N = 398	0.538
IV drug use	22 (15.7%)	54 (13.6%)	
Unprotected sex w/male	117 (83.6%)	340 (85.4%)	
Other risks	1 (0.7%)	4 (1.0%)	
**Baseline CD4**	N = 152	N = 459	**<0.001**
Median (cells/mm^3^)	352	246	
≤250 cells/mm^3^	50 (32.9%)	237 (51.6%)	
>250 cells/mm^3^	102 (67.1%)	222 (48.4%)	
**Baseline Viral Load (HIV RNA copies/ml)**	N = 146	N = 453	**<0.001**
<400	28 (19.2%)	47 (10.4%)	
400–9,999	54 (37.0%)	110 (24.3%)	
10,000–49,999	33 (22.6%)	100 (22.1%)	
50,000–99,999	13 (8.9%)	66 (14.6%)	
≥100,000	18 (12.3%)	130 (28.7%)	
**Hep C serology**	N = 109	N = 386	
Positive Hep C serology	24 (22.0%)	69 (17.9%)	0.328
Negative Hep C serology	85 (78.0%)	317 (82.1%)	

*Percentages may not equal 100 due to rounding.*

Patients on NVP-based regimens were significantly younger (mean: 32.8; SD: ±8.9 years) than those on non-NVP-based regimens (mean: 37.3; SD: ±7.9 years, p<0.001). **[** **Table 1** **]** However, when controlling for pregnancy there were minimal differences between groups. Specifically, in the non-pregnant group the mean (SD) ages for those receiving NVP-based regimens and non-NVP-based regimens were 38.8 (8.5) years and 38.6 (7.3) years, respectively. In the pregnant group, the mean (SD) age for NVP and non-NVP were 28.0 (5.5) and 29.4 (6.3), respectively. Pregnant women were more likely to be on NVP (86 [56.2%] vs non-pregnant 66 [14.4%] p<0.001), had higher CD4 counts (113 [74.3%] vs non-pregnant 210 [45.9.4%] p<0.001), and were of younger age (28.62 vs non-pregnant 38.68 p<0.001). Pregnant women were also less likely to have Hepatitis C (10 [9.2%] vs non-pregnant 83 [21.5%] p = 0.004). **[** **Table 2** **]** LFTs were assessed with equal frequency in the pregnant cohort and the non-pregnant cohorts (p = 0.388) as well as amongst those pregnant and not pregnant receiving NVP (p = 0.301) and non-NVP-based regimens (p = 0.712).

**Table 2:** Study Sample Demographic and Clinical Characteristics stratified by Pregnancy Status (N = 612).

	Pregnant (*n* = 152)	Not Pregnant (*n* = 460)	*P-value*
**Age (mean; years)**	28.62	38.68	**<0.001**
**Type of Regimen**	N = 153	N = 459	**<0.001**
NVP	86 (56.2%)	66 (14.4%)	
Non-NVP	67 (43.8%	393 (85.6%)	
**Race**	N = 153	N = 456	0.338
Black	117 (76.5%)	326 (71.5%)	
White, non-Hispanic	21 (13.7%)	90 (19.7%)	
Hispanic	14 (9.2%)	39 (8.6%)	
Multiracial	1 (0.7%)	1 (0.2%)	
**Mode of transmission**	N = 153	N = 459	**<0.001**
IV drug use	9 (5.9%)	67 (14.6%)	
Unprotected sex w/male	141 (92.2%)	316 (68.8%)	
Other risks	3 (1.9%)	76 (16.6%)	
**Baseline CD4**	N = 152	N = 458	**<0.001**
Median (cells/mm^3^)	401	236	
≤250 cells/mm^3^	39 (25.7%)	248 (54.1%)	
>250 cells/mm^3^	113 (74.3%)	210 (45.9.4%)	
**Baseline Viral Load (HIV RNA copies/ml)**	N = 148	N = 451	**<0.001**
<400	19 (12.8%)	56 (12.4%)	
400–9,999	76 (51.4%)	88 (19.5%)	
10,000–49,999	35 (23.6%)	98 (21.7%)	
50,000–99,999	13 (8.8%)	66 (14.6%)	
≥100,000	5 (3.4%)	143 (31.7%)	
**Hep C serology**	N = 109	N = 386	
Positive Hep C serology	10 (9.2%)	83 (21.5%)	0.004
Negative Hep C serology	99 (90.8%)	303 (78.5%)	

*Percentages may not equal 100 due to rounding.*

Among those receiving NVP, 20.8% were ART experienced and 70.0% were ART naïve and there were no significant differences in the demographics of ART experienced versus ART naïve women receiving NVP. Of those who were on NVP and pregnant, 43.0% were experienced and 53.0% were naïve and of those who were not pregnant, 57.0% were experienced and 47.0% were naive (p = 0.33). Amongst ART experienced women, 40.0% had CD4 ≤250 compared to 34.0% of those who were ART naive (p = 0.35).

Of those women on non-NVP based therapy, 18.3% (n = 83) were on nucleoside-based therapy only, though none received zidovudine monotherapy. Two hundred thirty four (50.9%) women were on protease inhibitors: 25.0% (n = 58) on lopinavir/ritonavir, 3.0% (n = 14) on amprenavir, 37.0% (n = 87) on nelfinavir, 1.5% (n = 7) on saquinavir, 5.0% (n = 23) on indinavir, and 9.8% (n = 45) on boosted or unboosted atazanavir. There were 143 (31.1%) women were on efavirenz, two of whom were pregnant.

Pregnant women on NVP started ART at a mean of 19.1 weeks gestation (median: 18 weeks, range 8–32 weeks). Women on non-NVP regimens started ART at a mean of 18.7 weeks gestation (median 16 weeks – range 3–38 weeks, p = 0.693). If women were started on ART during the prenatal period and thus had any exposure to ART during pregnancy, they were included in the pregnancy group. If they delivered during the 12–18 week period, we continued to follow them throughout the study time frame into the postpartum period.

**Adverse Events:** The majority of liver and skin AE in both regimen groups occurred within 6 weeks of initiating ART: 7/9(77.8%) ≥2 LEE on NVP, 16/26 (61.5%) ≥2 LEE non-NVP (p = 0.63 by continuity-corrected chi-square); 8/11 (72.7%) skin AE on NVP and 12/19 (63.2%) skin AE on non-NVP (p = 0.893 by continuity-corrected chi-square).

**Hepatic:** In total 38 subjects experienced LEE of grade ≥2 at baseline or during the 12–18 week follow-up period; 11 were excluded per exclusion criteria. There were no significant differences in LEE by regimen group: NVP 6/140 (4.3%) vs. non-NVP 21/459 (4.6%) (p = 0.885). [**Table 3**] Among women experiencing LEE, 7 developed grade ≥3 LEE, with no significant differences between NVP (n = 2, 1.4%) and non-NVP (n = 5, 1.1%; p = 0.668). Those on NVP with grade 3 LEE were pregnant with baseline CD4 >250 cells/mm^3^ (n = 2) and those on non-NVP were not pregnant with baseline CD4 ≤250 cells/mm^3^ (n = 5). **[** **Figure 1** **]**

**Table 3:** Clinical Characteristics of HIV+ women developing new onset grade ≥2 LEE (n = 27).

Characteristic	NVP-Regimen (n = 140)	non-NVP Regimen (n = 459)	Relative risk (95% CI)	*P* * *-value*
**All**	6/140(4.3%)	21/459(4.6%)	0.94 (0.38–2.28)	0.885
**Baseline (n = 599)**				
CD4≤250 cells/mm^3^	0/48 (0%)	16/237(6.8%)	0 (NA)	0.131
CD4>250 cells/mm^3^	6/92 (6.5%)	5/222 (2.3%)	2.9 (0.91–9.25)	0.122(a)
**Pregnant**	2/79 (2.5%)	0/67 (0%)		0.551
CD4≤250 cells/mm^3^	0/23 (0%)	0/16 (0%)	NA	NA
CD4>250 cells/mm^3^	2/56 (3.6%)	0/51 (0%)	NA	0.517
**Non-pregnant**	4/61(6.6%)	21/392(5.4%)		0.936
CD4≤250 cells/mm^3^	0/25 (0%)	16/221(7.2%)	0 (NA)	0.335
CD4>250 cells/mm^3^	4/36(11.1%)	5/171 (2.9%)	3.8 (1.07–13.46)	0.082
**HCV** ** **(n = 485)**	6/100(6.0%)	18/385(4.7%)	1.28 (0.52–3.15)	0.810
HCV Positive	2/22 (9.0%)	7/69 (10.1%)	0.9 (0.2–4.0)	1.000
HCV Negative	4/78 (5.1%)	11/316(3.5%)	1.47 (0.48–4.5)	0.737

*All p values are continuity-corrected chi-square, 2-tailed.

**HCV denominators are lower because this information was not available for all subjects.

*(a) p = 0.054 two-tailed for the comparison of NVP related relative risks in high and low baseline CD4 count groups.*

*NA: Risk estimates are not reported since adverse events were not observed for both treatment categories.*

There were no cases with initial grade ≥2 LEE at baseline who subsequently had higher liver elevations during follow-up. There were two women who had baseline liver elevations of grade 3 who did not progress and thus were not counted as LEE due to ART. In the univariate analysis, a non-significant trend was noted for an increased relative risk for the development of LEE in NVP users with baseline CD4 counts >250 cells/mm^3^ (p = 0.054 *two-tailed*). Patients on non-NVP regimes experienced a significant increase in LEE when the baseline HIV-1 RNA VL was greater than 100,000 copies/µl (p = 0.04), an association that was not noted in subjects on NVP-regimens.

Women who were co-infected with HCV were more likely than those without HCV to develop grade ≥2 LEE regardless of regimen group: 9.9% (9/91) of HCV co-infected patients vs. 3.8% (15/394) of HCV negative patients (p = 0.03). However, there was no difference in LEE between HCV patients on NVP (9.0%, 2/22) vs. HCV patients on non-NVP (10.1%, 7/69) (p = 1.0). In Hepatitis C negative patients, the relative risk for NVP verses non-NVP use and the development of grade >2 LEE in those with baseline CD4 >250 cells/mm^3^ was 5.81 (95%, CI: 1.1 to 30.8, p = 0.039 by Fisher's Exact Test). In contrast, no one in the NVP group with baseline CD4 ≤250 cells/mm^3^ developed LEE (p = 0.61 by Fisher's Exact Test, data not shown).

Multivariate logistic regression performed to identify independent predictors of new grade ≥2 LEE demonstrated an increased likelihood of hepatic AE in those with HCV co-infection (OR 2.502; 95% CI: 1.04 to 6, p = 0.040). Pregnancy status (OR 0.178; CI: 0.022–1.43, p = 0.104), NVP use (OR 1.62; CI: 0.55–4.76, p = 0.38) and baseline CD4 count >250 cells/mm^3^ (OR 0.62; CI: 0.25–1.50, p = 0.29) were not independently associated with the development of LEE.

**Adverse Events-Rash:** In total, 114/526 (21.7%) women developed a new rash (Grades 1–4) after therapy initiation with 30 (5.7%) women developing a new grade ≥2 rash during the study period. One non-pregnant subject on NVP was diagnosed with Stevens-Johnson syndrome 22 days after initiation of treatment; CD4 count at therapy initiation was 420 cells/mm^3^. The patient was hospitalized and had complete resolution of rash within 12 days of discontinuing ART. **[** **Figure 2** **]**

No difference in the frequency of new rash was seen between regimen groups. However, there was a higher incidence of severe rashes in the NVP regimen group vs. non-NVP group (p = 0.002): for grade 3 rash 2/127 (1.6%) on NVP-based regimens vs. 0/399 on non-NVP-based regimens; for grade 4 rash 2/127 (1.6%) on NVP vs. 0/399 on non-NVP. There was a non-significant trend towards a higher frequency of grade ≥2 rashes: 11/127 (8.7%) among those who initiated NVP-based regimens vs. 19/399 (4.8%) on non-NVP regimens (p = 0.099). **[** **Table 3** **]**

Furthermore, NVP-based regimens were significantly associated with the diagnosis of grade ≥2 rash in subjects with baseline CD4 >250 cells/mm^3^ (p = 0.001). A comparison of the relative risks for development of rash among women on NVP-based regimen was significantly different between the two CD4 sub-groups (p = 0.005), indicating an interaction between treatment regimen and CD4 count. There was a trend of increased risk for the development of grade ≥2 rashes in the NVP treatment group among pregnant women (p = 0.054) but not among non-pregnant women (p = 0.476).

Stratifying women by CD4 count at baseline, NVP exposure was associated with the development of grade ≥2 rashes in pregnant patients with baseline CD4 count >250 cells/mm^3^ (p = 0.042); a similar trend could be observed in non-pregnant patients (p = 0.058). No association with regimen use was seen with baseline CD4 count ≤250 cells/mm^3^.

Using multivariate logistic regression modeling controlling for type of regimen, pregnancy status and baseline CD4 count, women were more likely to develop a grade ≥2 rash if they initiated an NVP-based regimen (OR 2.78; 95% CI: 1.14–6.76, p = 0.024) or had baseline CD4 counts≥250 cells/mm^3^ (OR 2.68; 95% CI: 1.19–6.02 p = 0.017). Pregnancy status was not an independent predictor for the development of a grade ≥2 rash (OR 0.46 95% CI: 0.15–1.4p = 0.165). However, pregnant women were more likely to be on NVP and more likely to start therapy at higher CD4 counts. **[** **Table 4** **]**

**Table 4:** Clinical Characteristics of HIV+ women developing new onset grade ≥2 rash (n = 30).

Characteristic	NVP-Regimen (n = 127)	non-NVP Regimen (n = 399)	Relative Risk (95% CI)	*P* * *-value*
**All**	11/127 (8.7%)	19/399 (4.8%)	1.82 (0.89–3.72)	0.099
**Baseline (n = 526)**				
CD4≤250 cells/mm^3^	2/39 (5.1%)	17/189 (8.9%)	0.57 (0.14–2.37)	0.633 (a)
CD4>250 cells/mm^3^	9/87 (10.3%)	2/209 (1.0%)	**10.74 (2.37–48.7)**	**<0.001**
**Pregnant**	6/73 (8.2%)	0/64 (0%)	NA	0.054
CD4≤250 cells/mm^3^	0/20 (0%)	0/15 (0%)	NA	NA
CD4>250 cells/mm^3^	6/52 (11.3%)	0/49 (0%)	NA	**0.042**
**Non-pregnant**	5/54 (9.3%)	19/355 (5.7%)	1.63 (0.64–4.19)	0.476
CD4≤250 cells/mm^3^	2/19 (10.5%)	17/174 (9.8%)	1.08 (0.27–4.31)	1.000
CD4>250 cells/mm^3^	3/35 (8.6%)	2/160 (1.2%)	6.9 (1.20–39.77)	0.058

*All p values are continuity-corrected chi-square, 2-tailed.

*(a) p = 0.005 two-tailed for the comparison of NVP relative risks in high and low CD4 count groups.*

*NA: Risk estimates are not reported since adverse events were not observed for both treatment categories.*

A secondary analysis was performed to evaluate the AE patterns for CD4 percentage below and above 20%. During pregnancy there is a lowering of absolute CD4 count due to hemo-dilution and CD4 percentage measurements may be more reliable [13]. Our results demonstrated a non-significant trend in increased risk of LEE in women on NVP with a CD4 percentage above 20% (3/83, 3.6%) when compared to women on non-NVP-based regimens (1/188, 0.5%) (p = 0.164 RR = 6.8 (0.72–64.4). The risk of rash in those on NVP was increased five-fold among those with CD4 percent >20%, though this finding did not reach statistical significance (NVP - 4/79 (5.1%), non-NVP-2/178 (1.1%) p = 0.138, RR = 4.51 (0.84–24.1)). NVP use was also associated with an increased risk of rash in the low CD4 percentage group though to a lesser extent when compared to the group of individuals with higher CD4 percent values (NVP - 5/43 (11.6%), Non-NVP -17/211 (8.1%) p = 0.645 RR = 1.44 (0.56–3.7).

## Discussion

This large multi-center, retrospective cohort study of 612 pregnant and non-pregnant women on NVP and non-NVP ART regimens assessed the cumulative incidence of hepatic and rash-related adverse events over the first 18 weeks post-ART initiation. The strengths of this study include the multi-center nature of the study, the significant sample size and the use of a large non-pregnant comparison group, a group that has been missing from other studies evaluating the relationship of AEs and ART during pregnancy. The majority of liver and skin AEs in both regimen groups in the current study occurred within 6 weeks of initiating ART. There were no significant differences in the overall percentage of patients developing LEE between regimen groups. We did not find a statistically significant association between baseline CD4 cell count above 250 cells/mm^3^ and the risk of hepatotoxicity in patients taking NVP in univariate and multivariate analyses. Though a non-significant trend towards an increased risk of LEE in the higher CD4 count group was noted in the univariate analysis, no such association was noted in the multivariate analysis. NVP use in women with baseline CD4 counts ≤250 cells/mm^3^, including pregnant women, resulted in a hepatic safety profile similar to women treated with non-NVP regimens.

In comparison, both pregnant and non-pregnant women on NVP with a baseline CD4 count >250 cells/mm^3^ had a significantly increased rate of grade ≥2 rash compared to women on non-NVP regimens with CD4 count >250 cells/mm^3^. NVP use was predictive of rash when controlling for CD4 count at regimen initiation and pregnancy.

The lack of association of an effect of CD4 count on LEE is consistent with recent studies [9], [10], [14]. Ouyang reported on 1358 women with ART exposure during pregnancy and found no association between CD4 cell count above 250 cells/mm^3^ and risk of hepatotoxicity in patients taking NVP [9]. Our findings are also consistent with a large review on NVP and hepatotoxicity which also failed to demonstrate an association [14]. However, the results from this study are in contrast to prior published studies in which a greater proportion of women on NVP with CD4 counts >250 cells/mm^3^ had moderate to severe side effects as compared to those with CD4 counts ≤250 cells/mm^3^ [6]. Bersoff-Matcha found that both a higher CD4 at initiation of NVP therapy and a higher nadir CD4 count were strongly associated with the development of severe rash and discontinuation of therapy [15]. The risk of NVP–induced hepatitis was found to be increased 12-fold in women with greater than 250 CD4 cells/mm^3^ [16]. In a summary analysis of 17 clinical trials using NVP, the risk ratio was 9.8 in women with rash-associated hepatic events with CD4 count >250 cells/mm^3^ as compared to those with lower CD4 counts. [17].

In this study, pregnancy was not an independent risk factor for the development of LEE or rash. In contrast, a recent study of 2050 HIV-infected pregnant women concluded that NVP was not significantly associated with risk of LEE, and that pregnancy was a risk factor for LEE [10].

Hepatitis C co-infection was independently associated with the development of LEE in this cohort. This finding is consistent with previous studies that have examined the association of HCV with hepatoxicity in patients on ART. Co-infected patients in these studies were found to have a significantly greater risk of experiencing hepatic events [18]–[20]. Vogel and colleagues studied the impact of chronic viral hepatitis on the pharmacokinetics of NVP. They found that other factors such as accumulating NVP drug levels may be responsible for an increased risk of liver damage in HIV/HCV co- infected patients [21]. Rivero and colleagues found that HCV co-infection increased by two to seven fold the risk of developing LEE >2 in patients treated with non-nucleoside reverse transcriptase inhibitors [22]. Bonnett showed that patients with HCV and/or HBV co-infection who received NVP containing regimens had a 45% increase in hepatotoxicity at month 12 of follow-up when compared to patients without co-infection [23].

In our study, NVP use was predictive of rash even when controlling for CD4 and pregnancy. The 2NN Trial reported grade ≥3 rash occurrence in 3.4% of patients taking NVP twice daily [24]. Aggregate data from 1,752 patients who participated in 33 NVP clinical trials revealed a rash rate of 17.0%, the majority of which occurred within 6 weeks of NVP initiation: 6.0% discontinued NVP due to rash, and 0.5% developed Steven-Johnson syndrome [25]. Pollard reviewed prospective clinical trials with NVP and found an incidence of 0.3% SJS [26].

Recent studies have demonstrated that virologically suppressed patients switching to NVP do not show a higher risk of hepatotoxicity or rash dependent on CD4 counts [4]. The ATHENA cohort also suggested that the incidence of hypersensitivity reaction associated with NVP in patients with undetectable HIV RNA load at the start of NVP is lower in patients with prior treatment experience than those who are treatment naïve. While the importance of VL as a predictor for rash with NVP remains debatable, the current study has demonstrated that patients on non-NVP regimes had a significant increase in liver enzyme elevations at baseline HIV-1 RNA VL >100,000 copies/µl, a finding that was not seen in subjects on NVP-containing regimens (p = 0.04).

During pregnancy there is a lowering of absolute CD4 count due to hemo-dilution. The stability of CD4 percentage measurements in comparison to absolute CD4 counts between prepartum and postpartum periods have been confirmed [13]. Similar to absolute CD4 count, CD4 >20% in this study demonstrated a non-significant trend in the increase of LEE and rash-related events associated with the use of NVP.

Our study has limitations that should be considered. There was an overrepresentation of pregnant women who were using NVP, had higher CD4 counts, and were of younger age. These women were also less likely to have Hepatitis C. It could be hypothesized that the reason why LEE and rash were seen more frequently in women with higher baseline CD4 counts on NVP-based regimens was due to these confounding factors. However, pregnancy was not an independent predictor of LEE or rash. Since women in the United States who are HIV-infected and pregnant are many times diagnosed earlier in their disease process during pregnancy through HIV screening programs, and are younger due to the childbearing years, this overrepresentation is hard to avoid [27].

The limitation of a retrospective study resulted in the reliance on chart documentation to distinguish drug induced hepatotoxicity from hepatotoxicity due to other causes. Women who are pregnant have an increased rate of LEE due to conditions that are unique to pregnancy [10]. In addition due to insufficient reporting in clinic records, alcohol use, drug use, and Hepatitis B were not evaluated as confounding factors. The fact that a positive hepatitis serologic result was used as a surrogate for chronic active HCV infection may have resulted in an attenuation of the association between HCV and LEE as several of the women who were HCV antibody positive may have cleared their infection. Finally, AST/ALT values may not be specific enough markers for liver injury. It is known that only a small proportion of those with LEE will eventually develop fulminant hepatitis or acute liver failure and that LEE often resolve without any intervention. Many instances of drug induced liver disease are unpredictable and asymptomatic.

The results from this study should be considered in assessing the recommendations for use of NVP in all women, but particularly for women of childbearing age in resource limited settings (RLS). The 2009 World Health Organization (WHO) now recommends starting lifelong ART for all pregnant women with a CD4 count at or below 350 cells/mm^3^ regardless of symptoms. ART is recommended to be continued in all pregnant women during the breastfeeding period to reduce the risk of HIV transmission. These guideline changes mean that more pregnant women will be initiating NVP-based regimens as part of first-line ART therapy in RLS [28]. In comparison to other studies in RLS where an association between high CD4 counts and LEE and rash have been reported, this study supports increased confidence on the use of NVP in similar populations as pregnancy status, NVP use, and baseline CD4 count ≥250 cells/mm^3^ were not independent predictors for the development of LEE [28], [29]. In addition, the DART study showed that routine laboratory monitoring for toxic effects in HIV patients receiving ART had no benefit in RLS [30]. Our study confirms that while clinical monitoring would detect the increased risk of rash seen with NVP use in women with higher CD4 cell counts, the risk of LEEs described may not result in significant hepatotoxicity to necessitate lab testing. As rash and hepatic events occurred more frequently in the first 6 weeks of NVP initiation in this analysis, more diligent clinical monitoring is recommended during this time frame.

Our study cohort is representative of the HIV epidemic in women in U.S. women in terms of age, race, HIV transmission risk and HCV status, and therefore contributes to the knowledge needed to better characterize adverse events specific to this population (7). This large cohort study provides additional information for clinicians in assessing the risk of NVP-induced liver and skin toxicity by taking into account the short latency period and potential risk factors such as baseline CD4 counts and HCV co-infection. All HCV co-infected women on NVP based regimens should undergo close monitoring for LEE. Empiric use of NVP in the absence of resources to determine baseline CD4 count and monitor liver enzyme levels continues to call for careful consideration of the risks and benefits of NVP therapy. However, with the significant increase in women of childbearing age initiating NVP-based ART in RLS, where there is limited laboratory capacity to monitor for LEE, this study provides supportive evidence for the use of NVP in this population.
